# Cancer Risk Disparities between Hispanic and Non-Hispanic White Populations: The Role of Exposure to Indoor Air Pollution

**DOI:** 10.1289/ehp.0900925

**Published:** 2009-08-04

**Authors:** Diana E. Hun, Jeffrey A. Siegel, Maria T. Morandi, Thomas H. Stock, Richard L. Corsi

**Affiliations:** 1 University of Texas at Austin, Austin, Texas, USA; 2 University of Texas Health Science Center, School of Public Health, Houston, Texas, USA

**Keywords:** cancer risk assessment, formaldehyde, hazardous air pollutants, Hispanics, p-dichlorobenzene, personal exposure

## Abstract

**Background:**

Hispanics are the fastest growing minority group in the United States; however, minimal information is available on their cancer risks from exposures to hazardous air pollutants (HAPs) and how these risks compare to risks to non-Hispanic whites.

**Methods:**

We estimated the personal exposure and cancer risk of Hispanic and white adults who participated in the Relationships of Indoor, Outdoor, and Personal Air (RIOPA) study. We evaluated 12 of the sampled volatile organic compounds and carbonyls and identified the HAPs of most concern and their possible sources. Furthermore, we examined sociodemographic factors and building characteristics.

**Results:**

Cumulative cancer risks (CCRs) estimated for Hispanics (median = 519 × 10^−6^, 90th percentile = 3,968 × 10^−6^) and for whites (median = 443 × 10^−6^, 90th percentile = 751 × 10^−6^) were much greater than the U.S. Environmental Protection Agency (EPA) benchmark of 10^−6^. Cumulative risks were dominated by formaldehyde and *p*-dichlorobenzene (*p*-DCB) and, to a lesser extent, by acetaldehyde, chloroform, and benzene. Exposure to all of these compounds except benzene was primarily due to indoor residential sources. Hispanics had statistically higher CCRs than did whites (*p* ≤ 0.05) because of differences in exposure to *p*-DCB, chloroform, and benzene. Formaldehyde was the largest contributor to CCR for 69% of Hispanics and 88% of whites. Cancer risks for pollutants emitted indoors increased in houses with lower ventilation rates.

**Conclusions:**

Hispanics appear to be disproportionately affected by certain HAPs from indoor and outdoor sources. Policies that aim to reduce risk from exposure to HAPs for the entire population and population subgroups should consider indoor air pollution.

Evidence suggests that disparities in environmental exposures may disproportionately affect the health of ethnic minorities. Census tracts with higher proportions of Hispanics or African Americans appear to have higher outdoor levels of hazardous air pollutants (HAPs) than do tracts with higher proportions of non-Hispanic whites (Apelberg et al. 2005; Linder et al. 2008; Morello-Frosch and Jesdale 2006). However, this evidence is mostly based on outdoor measurements, and much less is known about exposure to indoor air pollution. Therefore, inhalation exposure assessments are needed to improve knowledge of environmental risk, given that these evaluations involve monitoring personal concentrations in the breathing zone of individuals throughout their daily activities. Such monitoring incorporates the penetration of outdoor pollutants into buildings, as well as important contributions from indoor sources of the HAPs and the large amount of time people spend indoors. The importance of indoor air to overall inhalation exposure is supported by results from various studies, most notably the Total Exposure Assessment Methodology (TEAM; Wallace 1991) and Relationships of Indoor, Outdoor, and Personal Air (RIOPA; Weisel et al. 2005) studies. These investigations demonstrate that some indoor sources can have greater effects on personal exposure to HAPs than those of outdoor origin.

Results from exposure assessments suggest that minority groups may have high exposures to specific HAPs that could cause significant disparities between these groups and the majority population. Pellizzari et al. (1999) used air pollutant data from the National Human Exposure Assessment Survey (NHEXAS) to determine that minorities had higher personal measurements for lead and benzene than did nonminorities, but the authors cautioned that their sample size for minorities was small. Churchill et al. (2001) analyzed exposure to volatile organic compounds (VOCs) through blood samples collected in the Third National Health and Nutrition Examination Survey (NHANES III) and indicated that African Americans and Mexican Americans were more likely to have elevated levels of *p*-dichlorobenzene (*p*-DCB) than were whites. African Americans also had higher blood levels of chloroform and tetrachloroethene than did whites. More recently, D’Souza et al. (2009) evaluated HAP data from the 1999–2000 NHANES and concluded that Hispanics and African Americans had much higher personal concentrations for BTEX (benzene, toluene, ethylbenzene, xylenes), methyl *tert*-butyl ether (MTBE), and *p*-DCB than did whites. However, NHANES did not evaluate exposure to carbonyls, building characteristics such as home ventilation rates, and cancer risks. The remaining investigations in the literature mostly provide insight on *p*-DCB and chloroform as possible pollutants of concern among minorities (Adgate et al. 2004; Sax et al. 2006).

In this study, we estimated the cancer risks of Hispanics and non-Hispanic whites due to exposure to HAPs using data from the RIOPA study. In RIOPA, nonsmoking residences in Los Angeles County, California (*n* = 105), Elizabeth, New Jersey (*n* = 100), and Houston, Texas (*n* = 106), were monitored. Approximately 48% of adult participants described themselves as Hispanic and 38% as white. We focused on 12 of the sampled airborne VOCs and carbonyls for which cancer unit risk factors are available and used personal concentrations to estimate contaminant-specific cancer risks and cumulative cancer risks (CCRs). We identified pollutants of most concern and explored their possible origins. We also investigated factors that could contribute to risk disparities by examining associations with demographic and building characteristics, because previous investigations reported that these could affect exposure to HAPs (Apelberg et al. 2005; D’Souza et al. 2009; Johnson et al. 2004; Linder et al. 2008).

## Materials and Methods

Data from the RIOPA study were made available by the Health Effects Institute (HEI 2008). The RIOPA study recruited nonsmoking adults who resided in Los Angeles County, California; Elizabeth, New Jersey; and Houston, Texas. Participants in Houston and Elizabeth constitute a convenience sample, whereas the Los Angeles participants were a subset from a randomly selected sample of individuals from another study. Approximately 100 adults volunteered in each city; most of these adults worked at home or at a workplace that was in the same neighborhood as their residences. About 65% of the homes were located in close proximity to major outdoor sources of pollution such as highways in Los Angeles, petrochemical facilities in Houston, and small sources, such as dry cleaners, in Elizabeth.

Weisel et al. (2005) has provided a detailed description of the field and measurement protocols used in the RIOPA study. Briefly, from 1999 to 2001, participants and their homes were monitored during two 48-hr periods that were approximately 3 months apart. Air contaminants were selected to include HAPs that are categorized by the U.S. Environmental Protection Agency (EPA) as urban air toxics or mobile-source pollutants, as well as compounds from primarily indoor origin. Air samples were collected concurrently in the personal or breathing zone, and inside and outside the house. Sixteen VOCs were monitored; using organic vapor monitors (OVM 3500, 3M Company, St. Paul, MN), and 10 carbonyls were collected using passive aldehyde and ketones samplers (Zhang et al. 2001). Concentrations at or below the respective method detection limit (MDL) were censored by replacement with half the MDL concentrations. The effects of censoring on the cancer risk assessment were small because at least 50% of the concentrations that contributed most significantly to risk were well above the MDL. Demographic and building characteristics, as well as daily indoor and outdoor activity patterns, were collected during each of the sampling sessions with questionnaires and walk-through surveys. Residential air exchange rates (AERs) were determined using tracer gas decay.

Cancer risks were used to evaluate the relative importance of sampled pollutants. Therefore, in this investigation we focused on twelve of the sampled HAPs for which estimates of cancer unit risk factors are available (Table 1). Risk factors were primarily obtained from the U.S. EPA (2005); however, estimates from the California Environmental Protection Agency (CalEPA 2005) and Caldwell et al. (1998) were used when not available from the U.S. EPA. Only houses with personal concentrations for all of these 12 compounds in either of the monitoring sessions were considered. The overall sample size was reduced from 311 to 243. Estimates of cancer risks for each HAP were derived as


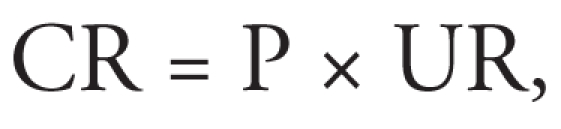


where CR is the cancer risk, P is the measured personal concentration (micrograms per cubic meter), and UR is the inhalation cancer unit risk factor and represents the probability of cancer for a 70-year exposure to 1 μg/m^3^. The CCR was calculated by summing the cancer risk from all 12 HAPs (Caldwell et al. 1998).

Several conventions were followed throughout this research. We excluded measurements from a household where someone smoked during a sampling period (*n* = 5). Information from the two sampling sessions was consolidated into a single data set. Air concentrations for each pollutant and AERs were averaged when the household was monitored twice. In most instances, demographic data from the first visit were selected when information from the first and second sessions were not in agreement. In the case of income, the midpoints of disparate income ranges were averaged.

We used nonparametric statistical analyses because pollutant concentrations typically had positively skewed distributions. The Wilcoxon rank-sum test was used to evaluate differences between two independent samples, such as personal concentrations from Hispanics and whites. Similarly, the Kruskal-Wallis test was used with three independent variables. The Wilcoxon sign-rank test was used to assess differences between paired samples, such as concurrent personal and indoor concentrations. Results were considered statistically significant at *p* ≤ 0.05. We used SPSS version 15.0 (SPSS Inc., Chicago, IL) for these analyses.

## Results

The CCRs for all participants in the RIOPA study (*n* = 238), including those who did not report to be Hispanic or white, exceeded 10^−4^. Mean, median, and 90th percentile CCRs were 1,126 × 10^−6^, 485 × 10^−6^, and 1,675 × 10^−6^, respectively, after excluding two unusually high measurements for chloroform (1,224 μg/m^3^) and tetrachloroethylene (1,340 μg/m^3^). The principal contributors to the mean CCR were *p*-DCB (60%) and formaldehyde (26%). For individuals with the highest risks (i.e., top 10th percentile), *p*-DCB accounted for 91% of the mean CCR.

### Differences between Hispanics and non-Hispanic whites

**Figure 1:**
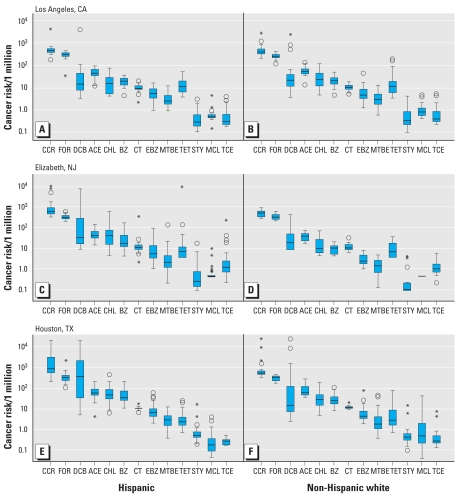
Distributions of cancer risks based on personal concentrations of Hispanics and whites in Los Angeles (*A* and *B*), Elizabeth (*C* and *D*), and Houston (*E* and *F*). Boxes represent 25th and 75th percentiles, whiskers indicate lower and upper range, and the black line is the median. Circles indicate values between 1.5 and 3 times the interquartile range. Asterisks demonstrate values > 3 times the interquartile range. Hispanic: Los Angeles, *n* = 23; Elizabeth, *n* = 54; Houston, *n* = 44. Non-Hispanic white: Los Angeles, *n* = 43; Elizabeth, *n* = 15; Houston, *n* = 36. Abbreviations: ACE, acetaldehyde; BZ, benzene; CCR, cumulative cancer risk; CHL, chloroform; CT, carbon tetrachloride; DCB, p-dichlorobenzene; EBZ, ethylbenzene; FOR, formaldehyde; MCL, methylene chloride; MTBE, methyl tert-butyl ether; STY, styrene; TCE, trichloroethylene; TET, tetrachloroethylene.

The percentage of Hispanic participants was the largest in Elizabeth (78%), followed by Houston (55%) and Los Angeles (35%) (Table 2). Figure 1 shows that for both Hispanics and whites, cancer risks for 9 of the 12 pollutants were higher than the U.S. EPA benchmark of 10^−6^, but differences in risk between the two ethnic groups varied by city. The CCR was higher among Hispanics than among whites in Elizabeth (*p* ≤ 0.05) and Houston (*p* ≤ 0.01). The median CCR of Hispanics in Elizabeth, 506 × 10^−6^, was 1.2 times higher than that for whites. This ratio increased to 1.6 in Houston where Hispanics had a median cumulative risk of 723 × 10^−6^. For Los Angeles, the CCR was about 438 × 10^−6^ for both ethnic groups and similar to that for whites in Elizabth and Houston. The main contributors to CCR were formaldehyde, *p*-DCB, acetaldehyde, chloroform, and benzene. These pollutants accounted for at least 83% of the cumulative risk among Hispanics and 92% among whites in each of the cities. The contribution among Hispanics increased to 95% after we excluded one unusually large tetrachloroethylene concentration in Elizabeth (1,340 μg/m^3^).

**Figure 2:**
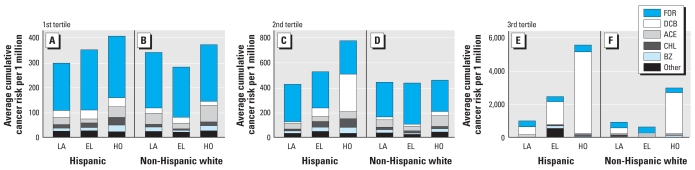
Average of 1st (*A* and *B*), 2nd (*C* and *D*), 3rd (*E* and *F*) CCR tertiles for Hispanics (*A*, *C*, and *E*) and whites (*B*, *D*, and *F*). Every tertile shows the average contribution of each HAP. Hispanic: LA, *n* = 23; EL, *n* = 54; HO, *n* = 44. Non-Hispanic white: LA, *n* = 43; EL, *n* = 15; HO, *n* = 36. Abbreviations: ACE, acetaldehyde; BZ, benzene; CHL, chloroform; EL, Elizabeth; FOR, formaldehyde; HO; Houston; LA, Los Angeles; Other, carbon tetrachloride, ethylbenzene, methylene chloride, MTBE, styrene, tetrachloroethylene, and trichloroethylene.

Given the skewed distribution of the CCRs in Figure 1, we analyzed the cumulative risk tertiles. Figure 2 shows the average contribution of each HAP to the mean of CCR tertiles. Most of the first and second tertiles for all of the studied scenarios were similar with mean cumulative values ranging from 281 × 10^−6^ to 524 × 10^−6^. Formaldehyde contributed 55–77% of the mean CCR, whereas acetaldehyde, benzene, chloroform, and *p*-DCB accounted for 18–36%. The second tertile for Hispanics in Houston differed by having a much higher mean value of 771 × 10^−6^, where *p*-DCB was the main contributor (39%) over formaldehyde (35%). Mean cumulative risks increased from the second to third tertiles more for Hispanics (factor of 2.3–7.2 across all cities) than for whites (factor of 1.3–6.4 across all cities). Increases in risk in Los Angeles and Elizabeth were primarily due to *p*-DCB, which accounted for approximately 53% of the mean CCR among Hispanics (CCR_Los Angeles_ = 969 × 10^−6^, CCR_Elizabeth_ = 2,437 × 10^−6^) and 28% among whites (CCR_Los Angeles_ = 889 × 10^−6^, CCR_Elizabeth_ = 604 × 10^−6^). In Houston, *p*-DCB was responsible for 88% of the mean CCR for Hispanics (CCR = 5,537 × 10^−6^) and 64% for whites (CCR = 2,964 × 10^−6^). Formaldehyde was the second most important pollutant for all of the third tertiles; it contributed 7–34% of the mean CCR among Hispanics and 10–60% among whites. The CCRs for Hispanics and for whites in the top two tertiles remained statistically different (i.e., Hispanics > whites) after we excluded *p*-DCB from the cumulative risk calculations in Elizabeth and Houston.

In Table 2, we summarize the personal concentrations for the two ethnic groups. Among the main contributors to the CCR, Hispanics in Elizabeth and in Houston had personal exposures that were statistically higher than those among whites for benzene, chloroform, and *p*-DCB. Whites in Los Angeles had statistically higher exposures than did Hispanics for acetaldehyde and chloroform, but these discrepancies were not large enough to cause statistical differences in CCR. Although formaldehyde did not contribute to risk disparities, it is worth noting that exposures were similar for all groups in all cities, which suggests chronic effects throughout the entire population. Formaldehyde had a mean and median personal concentration of 21 μg/m^3^ (cancer risk = 276 × 10^−6^), and the lowest coefficient of variance (28%) for all the evaluated HAPs after the exclusion of a relatively high personal measurement (144 μg/m^3^) that was inconsistent with the corresponding indoor concentration (26 μg/m^3^).

### Differences within Hispanics and non-Hispanic whites

We analyzed the demographic factors described in Table 3 to identify subgroups that may be at greater risk. We performed tests of statistical differences of cancer risks within ethnic groups. Because of the small sample sizes, we combined data from Los Angeles, Elizabeth, and Houston. The following results focus on the five HAPs that were the main contributors to CCR. The analysis indicates that Hispanic women had greater risk from exposure to chloroform (median = 36 × 10^−6^) than did Hispanic men (*p* ≤ 0.01), and their median values differed by a factor of 2.7. Hispanics who earned less than $25,000 had median risks for *p*-DCB (95 × 10^−6^) that were 4.1 times higher than those with greater incomes (*p* ≤ 0.01). Furthermore, Hispanics whose homes were less than 1 km from major outdoor sources of HAPs had median cancer risks for benzene (25 × 10^−6^) and *p*-DCB (110 × 10^−6^), as well as CCRs (642 × 10^−6^) that were statistically higher than those without these outdoor sources nearby. The apparent relationship between risk from *p*-DCB and proximity to outdoor sources is probably due to the prevalence of indoor sources of this pollutant in lower income homes. Fifty-eight percent of Hispanics who lived close to ambient sources had annual family earnings that were less than $25,000. Moreover, these individuals tended to have high personal concentrations of *p*-DCB, whereas major outdoor sources of this contaminant were not present. We observed no significant differences in cancer risk from demographic factors among whites.

Some of the building characteristics listed on Table 3 also influenced cancer risk. Just as with demographics, we pooled the data from the three cities for the two ethnic groups. We evaluated home age because it could be an indicator of the emission strength for certain HAPs from new building materials, as well as greater ventilation rates for older buildings. Hispanics whose houses were less than 15 years old had statistically higher median risks for acetaldehyde (48 × 10^−6^) and chloroform (51 × 10^−6^) than did those who lived in older homes. We observed similar trends among whites in newer homes for chloroform (40 × 10^−6^), benzene (21 × 10^−6^), and CCR (534 × 10^−6^). In general, we found a negative association between risk and the three AER ranges that we assessed (< 0.5, 0.5–1.0, > 1.0/hr). Hispanics in homes with low ventilation rates (< 0.5/hr) had statistically higher median risks for acetaldehyde (54 × 10^−6^), chloroform (61 × 10^−6^), and *p*-DCB (141 × 10^−6^), as well as CCRs (725 × 10^−6^), than did those in houses with high AERs (> 1.0/hr). The AERs were most influential on exposure to *p*-DCB, with a median risk ratio of 6.7 between homes with low and high AERs. Ventilation rates affected whites in a similar manner, with subjects in tighter houses (< 0.5/hr) having median cancer risk values for chloroform (24 × 10^−6^) and CCR (505 × 10^−6^) that were statistically higher than those in homes with AERs greater than 1/hr. There may have been some overlap between the positive associations of house age and ventilation rate and the risks from exposure to certain compounds generated indoors such as chloroform. This is consistent with the observed small increases in median AERs with building age. Nevertheless, house age may not be a good indicator of ventilation rate given that each of the studied age categories had comparable 5th and 95th percentile AER values.

### Sources of HAPs

We explored the possible origin of individual HAPs by examining statistical associations between personal and indoor concentrations and between personal and outdoor concentrations. Results from this evaluation are included in Table 2. In general, the analyses for both ethnic groups indicate that personal and indoor concentrations were similar and that personal concentrations were higher than outdoor concentrations (*p* ≤ 0.01). For most of the studied compounds, personal and indoor concentrations were probably influenced by the same source(s), and most of the exposure occurred indoors. In a few cases, personal concentrations were statistically higher than were indoor concentrations, which implies short episodic events where the participant may have been close to sources. Some exceptions to these observations included benzene and MTBE in Los Angeles, where personal and outdoor concentrations were statistically similar. Outdoor sources for these HAPs, particularly gasoline-powered vehicles, were likely dominant among participants in this city.

## Discussion

Few studies have examined cancer risks of minority groups from exposure to HAPs. We selected the Toxics Exposure Assessment Columbia-Harvard (TEACH) study (Sax et al. 2006) for comparison purposes because, except for 1,3-butadiene, both RIOPA and TEACH considered the same compounds and unit risk factors. The TEACH study evaluated mostly participants from minority backgrounds in New York City (NYC) (African American = 43%, Hispanic = 50%) and Los Angeles (LA_T_; Hispanic = 93%), although it only included high school students. We obtained mean (median) CCRs in Los Angeles, Elizabeth, and Houston among Hispanics of 556 (429), 962 (518), and 2,407 (699) per million, respectively. Comparable values of 957 (666) per million in NYC and 806 (486) per million in LA_T_ were reported in the TEACH study. Sax et al. (2006) also identified formaldehyde, *p*-DCB, acetaldehyde, chloroform, and benzene as the main contributors to CCR. *p*-DCB was responsible for the largest discrepancies in cumulative risk between and within TEACH and RIOPA.

We compared our observations on cancer risks from exposure to *p*-DCB, chloroform, benzene, formaldehyde, and acetaldehyde with those from four previous studies in the United States: the TEAM studies (Wallace 1991), which evaluated eight urban areas; NHANES III (Churchill et al. 2001) and 1999–2000 NHANES (D’Souza et al. 2009), which assessed the U.S. population; and NHEXAS (Clayton et al. 1999; Gordon et al. 1999), which examined six midwestern states. Both NHANES studies and NHEXAS used random and representative samples. To compare risks, we multiplied the personal concentrations reported in these investigations times the unit risk factors used in our analysis.

Our finding that Hispanics may be disproportionately affected by *p*-DCB is supported by results from TEAM and 1999–2000 NHANES. Cancer risks from exposure to *p*-DCB for Hispanics in RIOPA (mean = 899 × 10^−6^, median = 48 × 10^−6^), and in particular for those who resided in Houston (mean = 1,782 × 10^−6^, median = 305 × 10^−6^), were significantly greater than the estimates for the general population from the TEAM studies (mean = 242 × 10^−6^). Results from NHANES reinforce our observations because Hispanics from this investigation also had higher median cancer risks for *p*-DCB (52 × 10^−6^) than did whites (15 × 10^−6^). Common indoor sources of *p*-DCB include deodorizers, air fresheners, and moth repellents (Wallace 1991). These products are often pure *p*-DCB and are prone to relatively high mass emission rates. Answers to RIOPA questionnaires suggest that deodorizers and air fresheners are more prevalent among Hispanics than are moth repellents; 59% of Hispanics reported using air fresheners during the study, whereas only 6% used moth repellents. Solid toilet bowl deodorants may be of particular importance, as indicated by Churchill et al. (2001), whose analysis of data from NHANES III showed a 2-fold positive association between recent use of this type of product and increased blood levels of *p*-DCB. Serrano-Trespalacios et al. (2004) determined that toilet deodorants were present in 30% of the homes they monitored in Mexico City, but moth cakes were rarely found.

Chloroform also caused higher risks for Hispanics than among whites in the RIOPA and 1999–2000 NHANES studies. However, the risks we estimated for Hispanics were comparable to those from NHEXAS and TEAM for the general population. One of the problems with evaluating exposure to chloroform is that its main residential source is volatilization from chlorinated tap water, which has chloroform concentrations that are highly variable depending on the water source, date, and time. Median risks for Hispanics in Los Angeles were 1.7 times higher than those estimated by TEACH in this city. This discrepancy could have been influenced by differences in behavioral patterns between the participants in RIOPA (i.e., adults) and TEACH (i.e., high school students). Nevertheless, higher personal concentrations of chloroform among Hispanics may be because these households tend to exceed the U.S. average number of people per home by a factor of 1.35 (U.S. Census Bureau 2004), which may lead to a larger than average number of showers per residence. This could contribute to increases in cancer risks, because Nuckols et al. (2005) determined that chloroform concentrations in the blood and breath are affected by emissions that occur while others are taking showers. They also noted these increases in people who washed dishes by hand. Because this activity is usually performed by women, this could further explain our finding that Hispanic women had greater cancer risks for chloroform than did Hispanic men.

Benzene was also found to be a pollutant to which Hispanics may have higher exposures than do whites in the RIOPA and 1999–2000 NHANES studies. However, the median risk levels we estimated for Hispanics were comparable to or lower than those from 1999–2000 NHANES and NHEXAS (Clayton et al. 1999) for the overall population. This is probably because these two studies included participants who smoked whereas RIOPA did not, and smoking is the leading source of benzene in both personal and indoor air concentrations for the general population (Wallace 1996). Our results suggest that Hispanics may have a high risk from exposure to benzene because of the proximity of their homes to ambient sources of HAPs. However, in Elizabeth and Houston, their personal concentrations were statistically higher than outdoor levels, and no statistical differences were found between personal and indoor concentrations. Therefore, other sources close to the living areas, such as emissions from gasoline-powered devices, could have infiltrated indoors (Batterman et al. 2007) and affected the exposure of Hispanics to benzene. The role of gasoline is supported by high median exposures of Hispanics to MTBE, a VOC emitted exclusively by gasoline.

Formaldehyde was the largest contributor to CCR for 69% of Hispanics and 88% of whites. Moreover, both groups had a similar median risk for formaldehyde (276 × 10^−6^). Comparable values were estimated in the TEACH study (NYC = 222 × 10^−6^, LA_T_ = 266 × 10^−6^) and the NHEXAS pilot study in Arizona (273 × 10^−6^; Gordon et al. 1999). This consistency in estimated cancer risks suggests possible uniform chronic exposures to formaldehyde throughout the U.S. population due to prevalent indoor source such as pressed-wood materials. Acetaldehyde was among the important contributors to CCR for both Hispanics and whites. The TEACH study also reported acetaldehyde to be of significance with respect to CCR. For both RIOPA and TEACH the median personal and indoor concentrations of acetaldehyde were comparable, whereas median personal concentrations were 2 to 5 times higher than outdoor concentrations. Therefore, sources within residences were as important or more important than outdoor sources in terms of exposure and risk. Our evaluation suggests that combustion-related sources other than tobacco smoke, which was excluded from the RIOPA study, may have been of relevance because personal concentrations for acetaldehyde and benzene showed statistically significant correlations (Hispanics, Spearman coefficient *r_s_* = 0.22; whites, *r_s_* = 0.32). Similar results were observed with indoor concentrations (Hispanics, *r_s_* = 0.24; whites, *r_s_* = 0.30). Other possible indoor sources include detergents, cleansers and liquid wax (Nazaroff and Weschler 2004).

In general, Hispanics and whites who lived in houses with low ventilation rates had higher estimated cancer risks from exposure to HAPs, particularly from *p*-DCB and chloroform, which are consistent with results from TEACH (Sax et al. 2004). The cumulative effect of AER on exposure was demonstrated by statistical differences in CCR between participants who lived in homes with ventilation rates < 0.5/hr and > 1/hr. Moreover, higher median AERs in Hispanic households in Los Angeles (1.2/hr) than in Elizabeth (1.0/hr) and Houston (0.5/hr) may explain why *a*) Hispanics in Los Angeles had lower CCRs than did those in the other two cities, *b*) we observed no statistical differences in CCR in Los Angeles between Hispanics and whites (median AER for white households = 0.8/hr), and *c*) personal and outdoor concentrations for benzene and MTBE were statistically similar for Hispanics in Los Angeles. Differences in AER among cities may be because a larger percentage of Hispanic homes in Los Angeles (74%) reported to have had their windows open for some time during the sampling session than in Elizabeth (30%) and Houston (7%). Although these results suggest that ventilation rates can reduce risks from HAPs, this measure is not sufficient. People in homes with AERs 2.9 times higher than a recommended value of 0.35/hr (American Society of Heating, Refrigerating and Air-Conditioning Engineers 2004) experienced median CCRs of 435 × 10^−6^.

We compared our estimates with those from studies that were based on outdoor measurements. Morello-Frosch and Jesdale (2006) used ambient levels of HAPs from the 1996 National Air Toxics Assessment (NATA) and reported a mean CCR of 632 × 10^−6^ for the total population in U.S. metropolitan areas, and 900 × 10^−6^ for Hispanics. Another investigation, the Multiple Air Toxics Exposure Study III (MATES), evaluated outdoor contamination in California’s South Coast Basin. Measurements yielded a mean CCR of 1,200 × 10^−6^ (South Coast Air Quality Management District 2008). Mobile sources were important in both studies, accounting for approximately 88% of the CCR from NATA and 94% of the CCR from MATES. Diesel particulate matter contributed 53% and 84% of these CCRs, respectively. Our analysis included a subset of the HAPs that were evaluated in the other two studies, and these contaminants were predominantly of indoor origin. Nevertheless, our estimates of mean CCRs for Hispanics ranged from 556 × 10^−6^ to 2,407 × 10^−6^ and were either comparable to or greater than those from NATA and MATES.

Although the cancer risk assessment that we performed was a useful tool to place into context the measured personal concentrations from RIOPA in a standardized manner, the approach has limitations. Our calculations underestimate cumulative risk because we only analyzed 12 HAPs. Important contributors to cancer risk that were not part of our evaluation are polycyclic organic matter and 1,3-butadiene, for which Woodruff et al. (2000) estimated cancer risks of 72 × 10^−6^ and 31 × 10^−6^, respectively, using outdoor measurements. Other limitations include uncertainty in the derivation of cancer potency factors. Furthermore, cancer potencies assume 70-year lifetime exposures. Our estimates, like those of others reported herein, are based on a sample of this exposure. Additionally, the results of our evaluation should be considered with caution because the RIOPA participants were not selected using a random, stratified sampling scheme. Finally, the statistically significant discrepancies in CCR that we report between Hispanics and whites are primarily based on measurements from Elizabeth and Houston. As explained earlier, we observed no disparities in Los Angeles, likely because higher ventilation rates mitigate the effect of indoor sources. Despite these limitations, our analysis, together with results from prior studies, appear to provide compelling evidence for the assumption that air-pollutant–related cancer risk disparities between Hispanics and non-Hispanic whites are indeed likely, and substantiate the importance of the contribution from indoor air pollution to these risks.

## Conclusions

Median CCR for Hispanics and whites were two orders of magnitude greater than the EPA benchmark of 10^−6^. Risk estimates among the top 10th percentile of Hispanics were greater than 10^−3^. CCR for both ethnic groups was dominated by 5 of the 12 HAPs included in the study: formaldehyde, *p*-DCB, acetaldehyde, chloroform, and benzene. Exposure to all of these compounds but benzene was primarily dominated by indoor residential sources. Formaldehyde was the largest contributor to CCR for 69% of Hispanics and 88% of whites. Hispanics had higher exposures to some of these pollutants, leading them to have statistically higher CCR estimates than did whites. This outcome was mainly due to *p*-DCB, probably associated with the use of air fresheners that emit this VOC. Increases in house ventilation rate can decrease risks. However, our findings suggest that strategies to lower exposure to HAPs among groups that are at greater risk, as well as for the general population, should consider both improved ventilation and concurrent reductions in indoor sources of the HAPs included in this study.

## Figures and Tables

**Table 1:** Measured HAPs in RIOPA with available cancer unit risk factors.

Compound	WOE		Unit risk (per μg/m^3^)	Source^c^
	IRIS^a^	IARC^b^		
Acetaldehyde	B2	2B	2.2 × 10^−6^	1
Benzene	A	1	7.8 × 10^−6^	1
Carbon tetrachloride	B2	2B	1.5 × 10^−5^	1
Chloroform	B2	2B	2.3 × 10^−5^	1
Ethylbenzene	NC	2B	2.5 × 10^−6^	2
Formaldehyde	B1	1	1.3 × 10^−5^	1
Methylene chloride	B2	2B	4.7 × 10^−7^	1
MTBE	NC	3	2.6 × 10^−7^	3
*p*-DCB	NC	2B	1.1 × 10^−5^	3
Styrene	NC	2B	5.0 × 10^−7^	4
Trichloroethylene	NC	2A	2.0 × 10^−6^	3
Tetrachloroethylene	NC	2A	5.9 × 10^−6^	3

Abbreviations: IARC, International Agency for Research on Cancer; IRIS, Integrated Risk Information System; WOE, weight of evidence.

a IRIS classification: A, known carcinogen; B1 and B2, probable carcinogens; NC, not classified.

b IARC classification: 1, carcinogenic; 2A, probably carcinogenic; 2B, possibly carcinogenic; 3, not classifiable as to carcinogenicity to humans.

c Sources: 1, IRIS (U.S. EPA 2005); 2 and 3, CalEPA (2005); 4, Caldwell et al. (1998).

**Table 2:** Descriptive summary of personal concentrations (μg/m^3^), by city and ethnic group.

	Hispanic						Non-Hispanic white						
Compound	*n* (%)^a^	Mean ± SD	Median	%> MDL	P&I^b^	P&O^b^	*n* (%)^a^	Mean ± SD	Median	%> MDL	P&I^b^	P&O^b^	H&W^c^
Los Angeles													
Acetaldehyde	23 (61)	19.8 ± 8.58	17.7	100	I*	P**	43 (63)	24.9 ± 10.1	23.1	100		P**	W*
Benzene	23 (61)	2.31 ± 0.91	2.23	89			43 (77)	2.56 ± 1.32	2.33	87			
Carbon tetrachloride	23 (61)	0.59 ± 0.21	0.53	92		O*	43 (77)	0.61 ± 0.18	0.59	93		O**	
Chloroform	23 (61)	0.83 ± 0.76	0.58	76		P*	43 (77)	1.38 ± 1.29	0.90	87	I*	P**	W*
Ethylbenzene	23 (61)	2.29 ± 1.46	1.87	89		P**	43 (77)	2.30 ± 2.62	1.49	88		P**	
Fomaldehyde	23 (61)	21.4 ± 6.19	22.2	100		P**	43 (63)	21.7 ± 5.44	21.3	100	P*	P**	
Methylene chloride	23 (61)	1.33 ± 1.56	0.87	51		P**	43 (77)	1.63 ± 1.38	1.22	61		P**	W*
MTBE	23 (61)	12.7 ± 10.2	8.21	95			43 (77)	11.1 ± 7.84	8.75	99	P**		
*p*-DCB	23 (61)	16.5 ± 68.2	1.15	46		P*	43 (77)	12.1 ± 46.2	1.78	54		P**	
Styrene	23 (61)	1.04 ± 1.29	0.49	65		P*	43 (77)	1.04 ± 1.40	0.44	58		P**	
Tetrachloroethylene	23 (61)	2.30 ± 1.99	1.61	86		P*	43 (77)	3.81 ± 7.47	1.66	86		P**	
Trichloroethylene	23 (61)	0.31 ± 0.42	0.13	43			43 (77)	0.28 ± 0.39	0.13	45			

Elizabeth													
Acetaldehyde	54 (69)	18.6 ± 8.20	16.1	100	P**	P**	15 (67)	15.6 ± 6.71	14.9	100		P**	
Benzene	54 (81)	3.64 ± 4.21	1.93	78		P**	15 (87)	1.16 ± 0.59	1.15	57		P**	H**
Carbon tetrachloride	54 (81)	1.03 ± 2.64	0.64	94			15 (87)	0.74 ± 0.39	0.60	82			
Chloroform	54 (81)	3.18 ± 4.80	1.55	78	P**	P**	15 (87)	0.79 ± 0.82	0.39	54		P**	H**
Ethylbenzene	54 (81)	3.83 ± 6.20	1.89	81		P**	15 (87)	1.12 ± 0.72	0.85	64		P**	H**
Fomaldehyde	54 (69)	21.9 ± 5.82	20.8	100		P**	15 (67)	23.0 ± 7.54	21.4	100	P*	P**	
Methylene chloride	54 (81)	1.41 ± 2.53	0.84	9			15 (87)	0.84 ± 0.00	0.84	0			H*
MTBE	54 (81)	20.2 ± 60.0	7.16	93	P**	P**	15 (87)	5.94 ± 4.57	4.82	82			H*
*p*-DCB	54 (81)	44.1 ± 123	2.61	63		P**	15 (87)	4.54 ± 8.45	1.51	36		P**	H*
Styrene	54 (81)	1.89 ± 4.61	0.46	54	P*	P**	15 (87)	1.21 ± 2.32	0.17	29		P*	H*
Tetrachloroethylene	54 (81)	26.4 ± 182	1.06	44		P**	15 (87)	1.80 ± 1.55	1.01	57	P**	P**	
Trichloroethylene	54 (81)	3.35 ± 14.0	0.54	74		P**	15 (87)	0.73 ± 0.73	0.45	71		P*	

Houston													
Acetaldehyde	44 (64)	25.9 ± 14.5	21.9	97		P**	36 (39)	35.6 ± 24.8	23.0	100	I**	P**	
Benzene	44 (93)	5.77 ± 4.55	3.68	100		P**	36 (94)	3.46 ± 2.29	2.68	100	P*	P**	H**
Carbon tetrachloride	44 (93)	0.60 ± 0.10	0.58	99			36 (94)	0.66 ± 0.11	0.64	100		P**	W**
Chloroform	44 (93)	2.67 ± 2.81	1.70	96		P**	36 (94)	1.47 ± 1.38	1.02	89		P**	H**
Ethylbenzene	44 (93)	3.35 ± 3.54	2.21	100	P**	P**	36 (94)	2.79 ± 4.29	1.49	100	P**	P**	H*
Fomaldehyde	44 (64)	23.8 ± 19.9	20.7	97		P**	36 (39)	19.9 ± 4.75	20.8	100		P**	
Methylene chloride	44 (93)	0.77 ± 1.29	0.32	74	P*	P**	36 (94)	4.93 ± 12.9	0.89	87		P**	W**
MTBE	44 (93)	11.5 ± 8.78	9.54	98	P**	P**	36 (94)	16.9 ± 30.7	6.09	99		P**	
*p*-DCB	44 (93)	162 ± 312	27.7	84	I*	P**	36 (94)	75.5 ± 306	1.09	66	P*	P**	H**
Styrene	44 (93)	1.76 ± 4.20	0.88	92		P**	36 (94)	1.68 ± 4.32	0.74	87	P**	P**	H**
Tetrachloroethylene	44 (93)	0.57 ± 0.69	0.34	79	P**	P**	36 (94)	1.72 ± 2.87	0.40	80	P*	P**	
Trichloroethylene	44 (93)	0.12 ± 0.04	0.12	25		P*	36 (94)	0.27 ± 0.57	0.12	37	P*		

Abbreviations: I, indoor; MDL, method detection limit; MTBE, methyl *tert*-butyl ether.

a Number of participants (percentage of participants who were sampled twice).

b P: Personal concentrations were statistically higher than indoor or outdoor concentrations; I: indoor concentrations were statistically higher than personal concentrations; O: outdoor concentrations were statistically higher than personal concentrations.

c H: measurements were statistically higher among Hispanics than whites; W: measurements were statistically higher among whites than Hispanics.

* 0.01 < *p* ≤ 0.05;

** *p* ≤ 0.01.

**Table 3:** Selected characteristics of participants and households, by city and ethnic group [*n* (%)].^a^

	Los Angeles		Elizabeth		Houston	
Description	Hispanic	White	Hispanic	White	Hispanic	White
Sex						
Male	8 (63)	14 (71)	9 (78)	4 (75)	4 (75)	9 (33)
Female	15 (60)	29 (59)	45 (67)	11 (64)	40 (62)	27 (41)
Income ($US)						
< 25,000	7 (29)	8 (75)	23 (70)	4 (50)	30 (53)	5 (40)
25,000–49,999	4 (75)	17 (53)	6 (100)	3 (67)	10 (90)	14 (50)
50,000–74,999	10 (70)	7 (86)	5 (80)	4 (100)	3 (100)	10 (30)
> 75,000	2 (100)	11 (54)	3 (100)	3 (67)	0	6 (33)
Don’t know	0	0	13 (46)	1 (0)	1 (0)	1 (0)
Refused to answer	0	0	3 (67)	0	0	0
Building type						
Mobile/trailer	1 (100)	2 (50)	0	0	22 (50)	2 (0)
Single-family detached	15 (60)	22 (59)	9 (78)	7 (57)	20 (16)	34 (41)
Single-family attached	0	3 (0)	4 (50)	1 (100)	0	0
Apartment	7 (71)	16 (81)	38 (71)	7 (71)	2 (50)	0
Building age (years)						
< 5	4 (100)	12 (83)	2 (100)	0	4 (50)	0
5–15	0	2 (50)	4 (75)	0	7 (29)	7 (29)
> 15	17 (47)	29 (55)	24 (67)	14 (71)	24 (79)	27 (44)
Don’t know	2 (100)	0	24 (67)	1 (0)	9 (56)	2 (0)
Building AER (per hour)						
< 0.5	1 (0)	9 (100)	5 (80)	2 (100)	17 (53)	22 (73)
0.5–1.0	9 (56)	15 (80)	21 (90)	4 (50)	14 (71)	12 (83)
> 1.0	13 (62)	19 (95)	26 (81)	8 (62)	8 (62)	2 (50)
< 1 km from industry	3 (67)	6 (67)	20 (65)	9 (78)	42 (64)	32 (41)
Total participants	23	43	54	15	44	36

a Data are presented as number of participants (percentage of participants sampled twice).
